# Improving AoA Localization Accuracy in Wireless Acoustic Sensor Networks with Angular Probability Density Functions

**DOI:** 10.3390/s19040900

**Published:** 2019-02-21

**Authors:** Bart Thoen, Stijn Wielandt, Lieven De Strycker

**Affiliations:** KU Leuven, ESAT-DRAMCO, Ghent Technology Campus, 9000 Ghent, Belgium; stijn.wielandt@kuleuven.be (S.W.); lieven.destrycker@kuleuven.be (L.D.S.)

**Keywords:** acoustic localization, wireless sensor network, microphone array, angle-of-arrival (AoA), MEMS microphones, cross correlation, low-power devices

## Abstract

Advances in energy efficient electronic components create new opportunities for wireless acoustic sensor networks. Such sensors can be deployed to localize unwanted and unexpected sound events in surveillance applications, home assisted living, etc. This research focused on a wireless acoustic sensor network with low-profile low-power linear MEMS microphone arrays, enabling the retrieval of angular information of sound events. The angular information was wirelessly transmitted to a central server, which estimated the location of the sound event. Common angle-of-arrival localization approaches use triangulation, however this article presents a way of using angular probability density functions combined with a matching algorithm to localize sound events. First, two computationally efficient delay-based angle-of-arrival calculation methods were investigated. The matching algorithm is described and compared to a common triangulation approach. The two localization algorithms were experimentally evaluated in a 4.25 m by 9.20 m room, localizing white noise and vocal sounds. The results demonstrate the superior accuracy of the proposed matching algorithm over a common triangulation approach. When localizing a white noise source, an accuracy improvement of up to 114% was achieved.

## 1. Introduction

Developments in energy efficient electronic components have led to low-cost solutions that create opportunities for commercial, battery-powered Wireless Sensor Networks (WSNs) [1]. A useful application for these networks is indoor localization, enabling home assisted living [2,3], surveillance systems, etc. [4]. Current implementations often involve cameras to monitor the environment, requiring expensive hardware and high power to record, process, transmit and store the video stream [5]. Therefore, installations are generally invasive and building a battery powered system is still a challenge. The system presented in this article applies a different approach by using a battery powered Wireless Acoustic Sensor Network (WASN). In this case, the acoustic sensor nodes are deemed to be low power and low profile, resulting in a device that is easy to install and is able to operate for more than a year on a single battery. Compared to a camera oriented setup, the described WASN greatly reduces deployment and maintenance costs.

Common acoustic positioning techniques rely on energy, distance or direction measurements [6]. Energy based methods entrust on the diminishing sound intensity when the distance increases. While it is easy to obtain the intensity of the recorded sound, the technique strongly depends on the estimation of the path loss exponent. This parameter is crucial for the energy attenuation model [7]. Another way of finding the distances between sound source and the sensor nodes is by using Time Difference of Arrival (TDoA). This method relies on the finite propagation speed of acoustic waves to calculate relative distances based on time measurements. Therefore, an important prerequisite for TDoA measurements is the time synchronization between sensor nodes (e.g., < 30 μs for cm accuracy) [6]. TDoA based systems consist of fixed distributed sensor nodes (i.e., anchor nodes or beacons) with a single microphone, transmitting the recorded data to a central server. On this server, the data are correlated to find the TDoA, enabling the localization of the sound source with lateration techniques [6,8]. Directional based localization or Angle of Arrival (AoA) relies on the calculated angles of each sensor node to triangulate the position of the sound source. Instead of one microphone, an AoA sensor node requires a microphone array to determine the incident angle. The angular information can be processed on the node itself. Therefore, only angular information is transmitted to a central server. Compared to TDoA, where the complete audio stream is transmitted, the amount of data is significantly reduced. Since AoA localization relies on directional information, accurate time synchronization between nodes is not required. When localizing sparse and stationary sound events, millisecond accuracy is sufficient [6]. The independence of time synchronization combined with the transmission of only small data packets, consisting of angular information, will reduce the energy consumption of the wireless transceiver significantly [9]. Combined with a computationally efficient method of determining the angular information, the AoA technique is most suited for use on battery powered WASN.

Finding the AoA of acoustic sounds events is usually achieved using one of the following methods [6,10]. The first is the delay-based method where time differences between microphone signals are calculated using cross correlation [10,11]. The second method is Steered-Response Power (SRP) [10,12,13,14] and uses the delay-sum-beamformer method to steer the array over the spatial points of interest in order to get a distribution of the received signal power. The last method uses parametric algorithms such as MUltiple SIgnal Classification (MUSIC) or the more advanced Generalized EigenValue Decomposition-MUSIC (GEVD-MUSIC) [15] to improve the noise robustness. They depend on a computationally intensive eigenvalue decomposition [16] and therefore are not suitable for use on battery powered nodes. The first method on the other hand is the most computationally efficient [17], since it only relies on one single cross correlation between microphone pairs. Therefore, the delay-based AoA method is selected as the method of choice for a low-power wireless acoustic sensor node.

This study focused on activity based acoustic localization with minimal hardware requirements. The wireless acoustic sensor node consisted of a low-profile two-element microphone array with an inter microphone distance of 10 cm, limiting the framework to 2D source localization. When the on board activity detector triggered an acoustic event, the angular information of all the sensor nodes was collected at the central server. Here, a localization algorithm combined the results and pinpointed the location of the sound event. In this study, two localization algorithms were compared. The first one is a classic triangulation algorithm that calculates the least squares solution of all the received angles [8], resulting in a set of x- and y-coordinates. The localization accuracies of the triangulation algorithm were compared with the proposed approach. Instead of only transmitting the AoA, the resulting cross correlation function of the delay-based AoA algorithm was transmitted to the central server. The resulting cross correlation function acted as a Probability Density Function (PDF). Using these PDFs, a matching algorithm generated a Spatial Probability Density Function (SPDF) for each node in the room. The estimated position of the event was represented by the highest probability in the combined SPDFs. The matching was achieved by using a predefined dataset of equally distributed points in the room. One way of acquiring the points is to use a fingerprinting method [18,19], where prerecorded fingerprints are used as a reference dataset. Prerecording fingerprints is a labor-intensive task [19], therefore the matching algorithm presented in this article starts from a precalculated dataset of Line-of-Sight fingerprints distributed over a fine equally distributed grid.

The article is organized as follows. Section 2.1 starts with theory of time-delay AoA computation, followed by Section 2.2 where a thorough description of the localization algorithm is elaborated. Then, Section 3 presents a practical test setup, which was constructed to evaluate the accuracy of the different methods and algorithms. Next, the measurement results, analyses and comparisons are presented in Section 4. Finally, Section 5 formulates the conclusion of the acquired results.

## 2. Theory

### 2.1. Time-Delay Angle of Arrival Computation

An important advantage of the AoA localization method in WASNs is the limited amount of data that need to be transmitted to the central server. In its least complex form, only the angle suffices. Another benefit is that the nodes do not need accurate time synchronization, which means that the wireless transmission can be completely suspended when there is nothing to send. Due to the energy constraints of a battery powered node, the amount of calculations on the nodes needs to be reduced as much as possible.

The least computationally insensitive AoA algorithm is the delay-based method [20]. This technique calculates the time-lag between microphone signals, using cross correlation with or without weighting schemes, e.g., Generalized Cross Correlation with PHAse Transform (GCC-PHAT) [21]. The peak in the resulting correlation function is a measure for the time-lag $\Delta t_{ij}$ between microphone *i* and *j*. Following Equation (1), the AoA $\alpha_{\mathrm{ij}}$ is easily calculated, with $d_{\mathrm{ij}}$ denoting the distance between microphone elements and $v_{s}$ the speed of sound.

(1) $$\begin{array}{c} \sin\left(\alpha_{\mathrm{ij}}\right)=\frac{\Delta t_{\mathrm{ij}}\;\cdot \;v_{s}\;}{d_{\mathrm{ij}}} \end{array}$$

Usually, the cross correlation function $r_{\mathrm{freq}}$ is calculated in the frequency domain in Equation (3). $r_{\mathrm{freq}}$ is the inverse Fourier transform of the cross spectrum $R_{\mathrm{freq}}$, consisting of the complex conjugate of the Fourier transform of $s_{i}$ times the Fourier transform of $s_{j}$ in Equation (2). The peak in the correlation function results in the time-lag $\Delta t_{ij}$.

(2) $$\begin{array}{c} R_{\mathrm{freq}}=F{\left\{s_{i}\right\}}^{*}.F\left\{s_{j}\right\} \end{array}$$

(3) $$\begin{array}{c} r_{\mathrm{freq}}=F^{-1}\left\{R_{\mathrm{freq}}\right\} \end{array}$$

Frequency domain cross correlation automatically computes all correlation points for two input signals $s_{i}$ and $s_{j}$. The correlation function acquired from this method consists of 2N−1 correlation points, where *N* is the number of samples in each microphone channel. The useful correlation points in the cross correlation function $n_{s}$ strongly depends on the selected sample frequency $f_{s}$. In addition, the distance between microphone elements $d_{\mathrm{ij}}$ has a significant impact on this value.

(4) $$n_{s}=2\left\lceil d_{\mathrm{ij}}\frac{f_{s}}{v_{s}}\right\rceil +1$$

Equation (4) calculates the useful correlation points based on the previously described parameters. When working with low sample frequencies and small inter microphone distances, it becomes challenging to obtain a high value for $n_{s}$. When two input signals of 512 samples combined with a sample frequency of 32 kHz and an array with an inter microphone distance of 10 cm are used, then only 21 correlation points of the available 1023 are of interest.

A significant amount of processing time can be saved by only calculating these relevant cross correlation points. Time domain cross correlation can be used to solve this problem, because it is able to calculate a small number of correlation points in a computationally efficient way [20]. The time domain cross correlation is easily calculated as the dot product of the two input signals $s_{i}$ and $s_{j}$ [20]. The localization algorithm described in Section 2.2 requires not only the AoA, but a PDF over the entire array aperture. Because the probability function should be strictly positive, the input signals $s_{i}$ and $s_{j}$ are split up into positive (Equation (5)) and negative (Equation (6)) parts. $s_{i}^{+}$ are the positive values and $s_{i}^{-}$ are the negative values of $s_{i}$. Averaging the results of the two dot products will result in a strictly positive function, which is a measure for the PDF. To compute the cross correlation function, the input signal $s_{j}$ is zero-padded on both sides. Each cross correlation point *m* is calculated by shifting $s_{j}$ over $s_{i}$ for *m*-times. To correctly scale the resulting correlation function $r_{\mathrm{dotprod}}\left(m\right)$, each cross correlation value is divided by the number of overlapping zero-padded samples in Equation (7).

(5) $$r_{\mathrm{dotprod},\max}\left(m\right)=\sum_{n=0}^{N-1}s_{i}^{+}\left[n\right]\;\cdot \;s_{j}^{+}\left[n+m\right]$$

(6) $$r_{\mathrm{dotprod},\min}\left(m\right)=\sum_{n=0}^{N-1}s_{i}^{-}\left[n\right]\;\cdot \;s_{j}^{-}\left[n+m\right]$$

(7) $$\begin{array}{c} r_{\mathrm{dotprod}}\left(m\right)=\frac{r_{\mathrm{dotprod},\max}\left(m\right)+r_{\mathrm{dotprod},\min}\left(m\right)}{2} \end{array}$$

In this study, delay-based AoA with or without GCC-PHAT was evaluated. Since GCC-PHAT is defined in the frequency domain, where the cross spectrum $R_{\mathrm{freq}}$ of the standard cross correlation is divided by its absolute value, it results in the phase transformed cross spectrum $R_{\mathrm{freq},\mathrm{PHAT}}$ (Equation (8)).

(8) $$R_{\mathrm{freq},\mathrm{PHAT}}=\frac{R_{\mathrm{freq}}}{|R_{\mathrm{freq}}|}$$

To apply this method in the time domain, another approach is required. Instead of dividing $R_{\mathrm{freq}}$ by its absolute value in Equation (8), the PHAT weighting can be applied by whitening the input audio, as demonstrated by Van Den Broeck et al. [20], and can be achieved by using an adaptive Linear Prediction (LP) filter. The goal is to obtain a unity gain over all frequency bins, while not altering the phase. Applying this method to the sampled input audio and combining it with the previously described *dot product* cross correlation method will result in a time domain GCC-PHAT version of the cross correlation method. In this article it is further referenced as *dot product PHAT*.

### 2.2. 2D Localization Algorithms

In this paragraph, the two localization algorithms are elaborated. The first one uses a basic triangulation approach, while the second one represents the proposed solution. In this case the received correlation function of each sensor node is used as a PDF. Afterwards, starting from a precalculated dataset of Line-Of-Sight (LOS) PDFs, an SPDF is generated with the received PDFs of each sensor node using a matching algorithm [22].

#### 2.2.1. Triangulation

A straightforward way of location estimation using triangulation consists of calculating the least square solution of all the received AoAs ($\alpha_{1,\ldots ,Q}$) for a number of sensor nodes *Q* [8,23,24]. The estimated location $\hat{e}$ for all combined sensor nodes with coordinates $x_{1,\ldots ,Q}$ and $y_{1,\ldots ,Q}$ is calculated using:

$$\hat{e}={\left(H^{\prime }\;\cdot \;H\right)}^{-1}\;\cdot \;H^{\prime }\;\cdot \;c\;$$

where

$$H=\left[\begin{array}{cc} \sin(\alpha_{1}) & -\cos(\alpha_{1})\\ \sin(\alpha_{2}) & -\cos(\alpha_{2})\\ \vdots & \vdots \\ \sin(\alpha_{Q}) & -\cos(\alpha_{Q}) \end{array}\right]$$

and

$$c=\left[\begin{array}{c} x_{1}\sin\left(\alpha_{1}\right)-y_{1}\cos\left(\alpha_{1}\right)\\ x_{2}\sin\left(\alpha_{2}\right)-y_{2}\cos\left(\alpha_{2}\right)\\ \vdots \\ x_{Q}\sin\left(\alpha_{Q}\right)-y_{Q}\cos\left(\alpha_{Q}\right) \end{array}\right].$$

If a linear array is used, it is impossible to distinguish between angles impinging from the front or back of the array [8]. This problem is solved by placing the nodes close to the wall, eliminating the possibility of sounds arriving at the back of the array.

Using this method, small deviations in the AoA could have a big impact on the localization error. When the number of sensors is limited, finding stray angles is difficult [25]. Because the method only relies on the single transmitted AoA value, much information regarding possible reflections or the magnitude of the correlation peak is lost. By transmitting the calculated cross correlation function in Equation (7), more information is available and can be exploited as described in the next paragraph.

#### 2.2.2. Matching Algorithm

**Figure 1:**
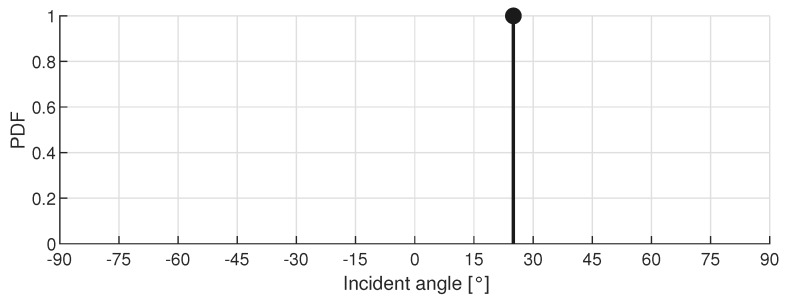
Example of an $f_{\mathrm{LOS},i}$-PDF with incident angle 26 stored in the reference dataset T.

If the central server receives the complete correlation function instead of the AoA of each node, more information is available and a matching algorithm can be used. The incoming data of each sensor node is matched to a reference dataset, similar to common fingerprinting localization systems. The matching algorithm starts from the room dimensions and generates a precalculated reference dataset T, consisting of the actual Line-Of-Sight (LOS) PDFs ($f_{\mathrm{LOS},i}$) for a number of reference points $N_{f}$. The locations are equally distributed along a rectangular grid in the room. A similar dataset exists for every sensor node in the room. Each LOS-PDF contains 181 values, representing an array aperture from -90 to 90 with a resolution of 1. At the position of the actual angle the value is set at 1, while the remaining points are set to 0. For example, Figure 1 depicts an $f_{\mathrm{LOS},i}$-PDF when the actual AoA is 26.

To match a measured PDF with each reference point $f_{\mathrm{LOS},i}$ an interpolation of the measured correlation function is required. The data are interpolated using cubic spline interpolation over the same angular resolution as the PDFs in the reference dataset, meaning 181 values over a range of -90 to 90. The resulting PDF is referenced as m in the following equations. The actual matching of $f_{\mathrm{LOS},i}$ and m is performed by calculating the Pearson correlation coefficient $r_{\mathrm{corr}}\left(i\right)$ for the $N_{f}$ reference points:

$$r_{\mathrm{corr}}\left(i\right)=\frac{\mathrm{cov}(f_{\mathrm{LOS},i},m)}{\sqrt{\mathrm{var}\left(f_{\mathrm{LOS},i}\right)\;\cdot \;\mathrm{var}\left(m\right)}}.$$

The resulting coefficients are then scaled linearly to an actual, strictly positive, SPDF ($r_{\mathrm{SPDF},\mathrm{corr}}\left(i\right)$) using:

$$r_{\mathrm{SPDF},\mathrm{corr}}\left(i\right)=\frac{r_{\mathrm{corr}}\left(i\right)+1}{N_{f}+\sum_{i=1}^{Nf}r_{\mathrm{corr}}\left(i\right)}.$$

**Figure 2:**
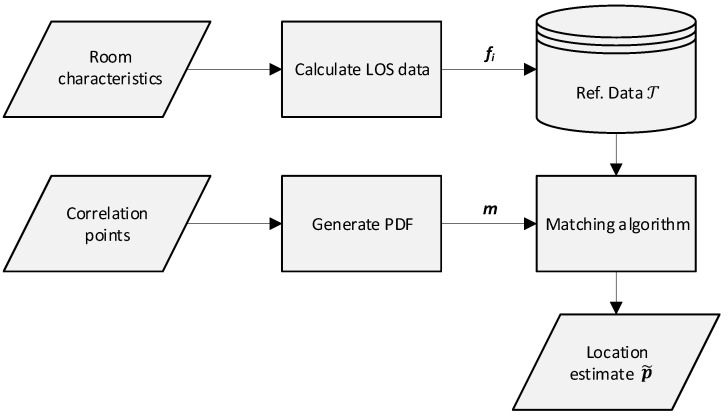
Flowchart of the PDF matching algorithm localization solution.

The SPDF represents the correlation between m and each point in the reference dataset T. If multiple sensor nodes are present in the environment, the SPDFs can be combined linearly with equal weights using:

$$r_{\mathrm{SPDF}}\left(i\right)=\frac{1}{Q}\sum_{q=1}^{Q}r_{\mathrm{SPDF},q}\left(i\right),$$

where *Q* denotes the number of nodes. The grid point with the highest value in $r_{\mathrm{SPDF}}\left(i\right)$ is selected as the estimated location $\tilde{p}$, resulting in a set of x- and y-coordinates. The flowchart in Figure 2 summarizes the operation of the localization solution.

## 3. Test Setup

**Figure 3:**
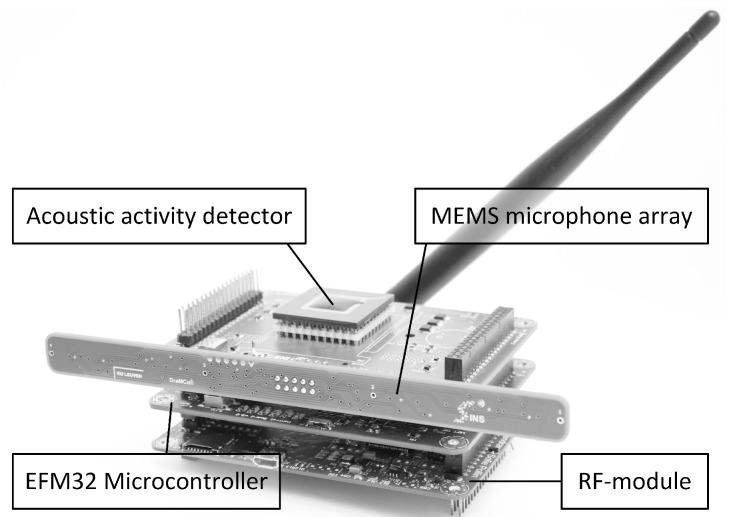
Picture of the WASN-node.

The test setup consists of several distributed WASN-nodes, depicted in Figure 3. The nodes feature a microphone array, wireless transceiver and microcontroller. During an acoustic event, the node samples the acoustic signals, calculates the correlation points and wirelessly transmits the resulting correlation points to a central server. The central server uses the received correlation points as an input for the matching algorithm and triangulation algorithm.

### 3.1. WASN-Node

**Figure 4:**
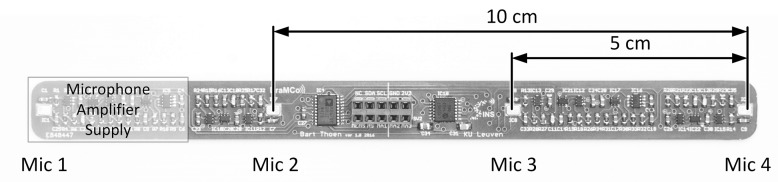
Picture of the linear microphone array with four MEMS microphones spaced 5 cm apart [27].

The node only wakes on an acoustic event, therefore a dedicated Acoustic Activity Detector (AAD) is present in the system. The AAD is always powered and activates the necessary components when sound is detected [26]. On an event, an EFM32 ARM cortex M4 microcontroller from Silicon Labs is activated. On its turn, the microcontroller wakes the linear microphone array, which is depicted in Figure 4 [27]. The array contains four omnidirectional MEMS microphones with each element having its own dedicated amplifier. The distance between microphone elements is limited to keep the WASN-sensor size acceptable for everyday applications [27]. In this study, only two of the four microphones were used to reduce the power consumption to an absolute minimum. A microphone distance of 10 cm was selected because it easily fits in a handheld device. The sampling of audio signals began after activating and stabilizing the amplifier outputs, directly followed by the processing of acoustic data. The previously described hardware is limited to a sampling rate of 32 kHz, when operating in its most energy efficient configuration. Using this configuration, the number of usable correlation points ($n_{s}$) is 21. Per acoustic event, 1024 samples are recorded. The microcontroller generates the cross correlation function using *dot product* and *dot product PHAT* cross correlation methods. The resulting 21 correlation points are wirelessly transmitted to the central server, using an IEEE 802.15.4 standard compliant radio. To save valuable energy, the node returns to sleep and waits for the next acoustic event. Table 1 summarizes the time and energy consumption of the WASN-node for each step in the process. When evaluating the energy values of the sensor node, it is clear that the majority of the energy consumption is assigned to the wireless communication. If all the sampled data were transmitted to the server, the energy consumption of transmission per event would be 124.6 mJ. Compared to the energy required for processing, 0.283 mJ for *dot product* and 0.614 mJ for *dot product PHAT*, it is clear that processing the data on the board itself is more efficient than transmitting the sampled audio. The energy required to transmit a single AoA value compared to the energy required to transmit the calculated correlation function is only 8% higher and mainly caused by the overhead of activating and connecting the wireless module [9].

The localization algorithm on the server processes the received data of all the nodes. First, the data are interpolated over 181 values using cubic spline interpolation, representing the total aperture range from -90 to 90 with a resolution of 1. For the triangulation algorithm, the peak in the interpolated PDF determines the time difference $\Delta t_{ij}$ and using Equation (1) results in the AoA of each sensor node. In the case of the proposed solution, the complete measured correlation function of each node is processed by the matching algorithm.

**Figure 5:**
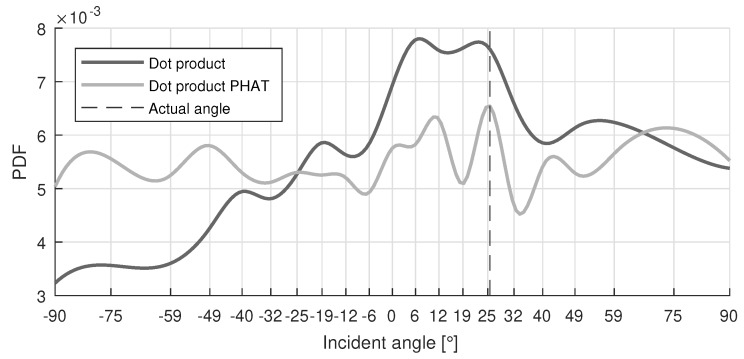
Example of an acoustic PDF with incident angle 26 for WN sound events, where *dot product PHAT* shows less pronounced peaks.

Figure 5 visualizes the PDF, with an incident angle of 26, for the *dot product* and *dot product PHAT* correlation methods. In this figure the main difference between the two methods can be observed: the PHAT method generates sharper peaks, but for the depicted incident angle the intensity of the direct wave is less pronounced than the other peaks in the PDF. The x-axis also shows the angular distribution of the calculated correlation points. This is caused by the sine term in Equation (1). The density of correlation points decreases with increasing incident angle, resulting in larger errors when the incident angle increases.

### 3.2. Measurement Environment

**Figure 6:**
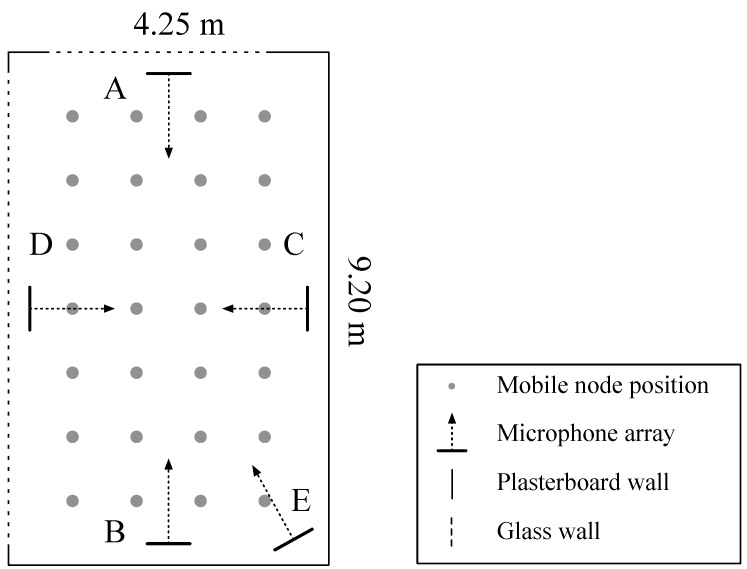
Layout of the practical test setup with five sensor nodes A, B, C, D and E.

To test the accuracy of the two algorithms, five sensor nodes (A, B, C, D and E), containing the previously discussed hardware, were deployed in an empty rectangular meeting room. Figure 6 depicts the setup, being 4.25 m wide, 9.20 m long and 3.5 m high. Most of the walls are made of plasterboard, while the wall behind A and D contains single sided glass. Table 2 summarizes the RT60 reverberation time of the room, measured with an NTI XL2 acoustic analyzer with M2211 measurement microphone. Arrays A, B, C and D are positioned parallel to and in the middle of each wall, at a distance of 19 cm from that wall and at height of 1.55 m. Array E is positioned perpendicular to the room diagonal at the same height. An equally distributed grid of 28 positions (gray dots in Figure 6) was used for test measurements. Each position was evaluated by playing two sound samples with a different bandwidth, namely white Gaussian noise and female spoken Harvard sentences [28]. The white Gaussian noise signal had a bandwidth of 48 kHz, while the audio file of the female spoken sentences only had a bandwidth of 4.3 kHz. The sound events were played through a dodecahedron omnidirectional speaker at a height of 1.55 m. The speaker produces a spherical acoustic wave, excluding directional effects of the localized sound source. The matching algorithm started from a reference set T consisting of 42 points along the width and 92 points along the length of the room, resulting in an equally distributed grid of 3864 points for each node. The reference dataset was precalculated during the setup phase of the localization system, requiring 13 s of processing time for each sensor node in the system. The data were generated using a Matlab script, running on machine with an Intel Core i7-2600 processor. After an acoustic event, the recorded data were received and processed by another Matlab script generating the SPDF for each sensor node. It took 2.1 s to compute the SPDF of each sensor node, but this could be reduced significantly by using optimized compiled code.

**Figure 7:**
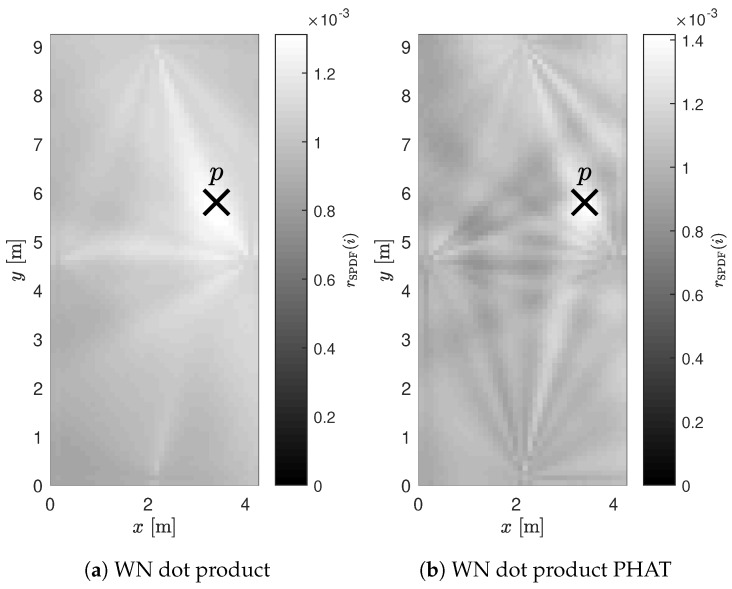
Example of the merged SPDFs for four nodes in a rectangular room where p is the actual position of the sound source.

Figure 7 visualizes two merged SPDF maps for four nodes placed in the center of each wall. The first SPDF was generated for the *dot product* method, while the second one used the *dot product PHAT* method. A light color in the figure means a high location probability.

## 4. Localization Results

### 4.1. Evaluation of the Localization Error

To evaluate the performance of the localization algorithms, the localization error $\epsilon_{\mathrm{loc}}$ between the actual position of the sound source p and the estimated position $\tilde{p}$ was calculated as the Euclidean distance. Because this value strongly depends on the dimensions of the test environment, the results were normalized by dividing the error value by the room diagonal, resulting in a normalized localization error ${\hat{\epsilon }}_{\mathrm{loc}}\leq 1$. This value made it easier to compare accuracy results between rooms of different sizes.

By relying only on ${\hat{\epsilon }}_{\mathrm{loc}}$ values, it was impossible to evaluate localization performance for only one sensor node. Therefore, we introduced the “Surface Interval” (SI) [22], defined in Equation (9). This is a dimensionless quantity between 0 and 1 that represents the percentile of the SPDF that contains the real position p. The value determines the fraction of the room surface that needs to be isolated to contain the p. A value close to zero means a high accuracy. The SI parameter is employed to evaluate single array results, since localization errors cannot be determined in a single array system. An SI value close to zero means the localization method is able to accurately separate the real position p in the room. A 50th percentile (P50) SI value >0.5 or a 95th percentile (P95) SI value >0.95 value implies that the localization method is unable to provide information on the real position p of the sensor node.

(9) $$\begin{array}{c} SI=P(r_{\mathrm{SPDF}}\left(i\right)>r_{\mathrm{SPDF}}\left(p\right)) \end{array}$$

The SI and ${\hat{\epsilon }}_{\mathrm{loc}}$ results are summarized in Table 3, Table 4, Table 5 and Table 6 listing the mean error, P50 and P95. These numerical parameters are commonly used for the assessment of localization accuracy, providing insight to the distribution without plotting.

### 4.2. Evaluation of the Matching Algorithm

#### 4.2.1. Individual Array Locations

The first results, summarized in Table 3, assess the SI values of the different individual array locations with WN recordings.

The *dot product* results indicate that placing the array in the center of the short wall led to the best overall results. Mean SI values of 0.08 were found for arrays located at the short wall, while arrays at the long walls had a mean SI value of 0.11. The increase in SI for arrays placed at the longer wall can be explained by the reduced accuracy at larger incident angles (-90 and 90), which was caused by the lower amount of correlation points in that area, as shown in Figure 5. For the arrays placed against the longer wall, the impinging angles were distributed more towards the larger incident angles and consequently generated less accurate results. A logical assumption in counteracting this effect was limiting the field of view, e.g., by placing the array in a corner of the room. Table 3 also lists the results for this configuration. Unlike what would be expected, the results are clearly less accurate, e.g., mean SI values of 0.35. Because the array was placed in a corner, propagation effects deteriorated the AoA estimation performance. The angular window was also reduced, decreasing the number of useful correlation points even further.

The *dot product PHAT* approach performed differently. The arrays on the short walls still had the lowest value, but they were slightly higher than the *dot product* method. For the long wall, similar results were noted. The main difference was found in the P50 and P95 values: the P50 value of the arrays on the long wall (0.039) was significantly lower than the *dot product* value (0.061). This can be explained by the fact that the *dot product PHAT* method performed best for positions close to the array. However, P95 values demonstrated the downside of this method. While performing really well for positions close to the array, SI values significantly increased for locations further away. The SI values from Array E were significantly better compared to the *dot product* method. The explanation for this difference was found in the shape of the PDF. While the angular window was smaller, the *dot product PHAT* method produced more pronounced peaks in the LOS direction. This led to more distinct peaks in the SPDF compared to the *dot product* results, as demonstrated in Figure 7.

Overall, the SI results of Array E are significantly higher for both methods, therefore this array was omitted in the following comparisons.

#### 4.2.2. Different Array Pairs

The SI and ${\hat{\epsilon }}_{\mathrm{loc}}$ results of three different pairwise array combinations were combined and compared: the arrays at the long walls (CD), the arrays on the short walls (AB) and the arrays placed at each adjacent wall (orthogonal combinations AD, BD, BC and AC). The results for white noise recordings are listed in Table 4. Because the results of two nodes were combined, a decrease of 50% was expected in the SI values. Looking at the mean SI and P50 SI values, this statement was confirmed; for most values, the decrease was even larger. Looking at the normalized errors of the *dot product* method, the arrays on the short wall resulted in the most accurate localization, closely followed by the orthogonal arrays, with mean normalized location errors of 0.1. The positioning accuracy for arrays on the long wall was the lowest, reason being the less pronounced peaks in the *dot product* PDF at incident angles higher than ±45.

Comparing the previous results with the *dot product PHAT* results indicated a clear difference. The arrays placed against the long walls resulted in the smallest localization errors, with a mean ${\hat{\epsilon }}_{\mathrm{loc}}$ error of 0.08 (i.e., a mean $\epsilon_{\mathrm{loc}}$ of 79 cm). The method also resulted in larger errors on the short and orthogonal walls. Especially the P95 value increased drastically, as the distance between sound source and array had a vast influence on the localization accuracy. This explains the excellent results for the nodes on the long walls for the this method: all measured locations in the middle of the room were positioned closest to both arrays.

#### 4.2.3. Multiple Array Combinations

To finalize the evaluation of the matching algorithm, ${\hat{\epsilon }}_{\mathrm{loc}}$ results of all array combinations (A, B, C and D) are listed in Table 5. To evaluate the ${\hat{\epsilon }}_{\mathrm{loc}}$ accuracy for recordings with fewer spectral components, female voice recordings, referenced in the table as “voice”, were added. The WN results of the two array combinations are obviously the same as in those discussed above. The *dot product* results show a decrease of 37% in mean localization error when adding an extra array to combination A and B. At the same time, the P95 also dropped by 33%. Adding a third array to nodes C and D showed less promising results: the mean error decreased, but the P95 value remained the same. The latter configuration could not improve on the outliers, because the added node only improved the localization accuracy on its side of the room. Comparing the results of four arrays against the three-array (ABC and ABD) combinations, only a slight improvement on the accuracy could be observed. This proved that the two dominant array positions for *dot product* were A and B, and adding one array on a long side of the wall gave the same results as combining data from four arrays.

Looking at the WN results of the *dot product PHAT* method, the difference in accuracy between the two triple array options was similar and can be explained by the overall superior performance of array combination C and D. Comparing the triple array errors to the best *dot product* triple array errors demonstrates that *dot product PHAT* performed equally well. The main improvement was found when adding the fourth array to the equation. Here, the *dot product PHAT* method outperformed all other results with a mean ${\hat{\epsilon }}_{\mathrm{loc}}$ of 0.04 and a P95 value of 0.12, resulting in a mean $\epsilon_{\mathrm{loc}}$ of 40 cm and an absolute P95 of 126 cm. The column on the right summarizes the ${\hat{\epsilon }}_{\mathrm{loc}}$ results for vocal sounds. First, the mean error values for vocal sounds were at least twice as high as those of the WN results. Evaluating the number of combined arrays using the *dot product* method led to the same conclusions as the WN results, the only difference being the increased error values. The four-array configuration performed best, but marginally better than using only three arrays. The mean $\epsilon_{\mathrm{loc}}$ of four arrays with voice recordings was 191 cm with an absolute P95 value of 338 cm, which was significantly worse than the WN results.

For the *dot product PHAT* method, different conclusions could be drawn for vocal sounds. While the mean ${\hat{\epsilon }}_{\mathrm{loc}}$ exhibited comparable results to the *dot product* method, a significant difference was observed in the P95 error values. For example, when we compared the results of the four-array setup, the *dot product* method had a P95 value of 0.38 compared to the *dot product PHAT* value of 0.58, resulting in a 53% increase. The conclusion of this example is that the outliers would be significantly higher when using the matching algorithm in combination with the *dot product PHAT* method for vocal sounds.

### 4.3. Comparison of the Proposed Algorithm to Triangulation Based Positioning

The matching algorithm was then compared with the classic triangulation algorithm. The resulting x- and y-coordinates from the least squares solution were not confined within the boundaries of the room, therefore errors larger than the room diagonal were possible. A summary of the localization results is given in Table 6, which includes the same array combinations as Table 5. The WN *dot product* results indicate that the matching algorithm clearly outperformed the triangulation algorithm, with a mean $\epsilon_{\mathrm{loc}}$ of 58 cm compared to 85 cm when localizing with four arrays, resulting in a localization improvement of 37% for the proposed solution.

The matching algorithm using the *dot product PHAT* method, achieved a mean $\epsilon_{\mathrm{loc}}$ of 40 cm while the triangulation algorithm had a mean $\epsilon_{\mathrm{loc}}$ of 146 cm, presenting a 114% performance improvement. The P95 values of the *dot product* method were slightly higher than those of the matching algorithm. On the other hand, the *dot product PHAT* method showed a significant increase in P95 values resulting in absolute localization errors of 400 cm. The matching algorithm clearly outperformed the triangulation algorithm for WN, particularly for the *dot product PHAT* method. When localizing vocal sounds, the matching algorithm performed 5% better for *dot product* and 12% for *dot product PHAT*.

### 4.4. Summary of the Results

This final paragraph gives a brief interpretation of the previously described results. It is clear that the matching algorithm, compared to the common triangulation algorithm, performed best for all localization results, certainly for WN sounds. Focusing on the matching algorithm, two different calculation methods were compared: *dot product* and *dot product PHAT*. The *dot product* algorithm exhibited the overall best performance. On some occasions, *dot product PHAT* had better results, because the accuracy was better in the close vicinity of the arrays, e.g., WN with four arrays. If the *dot product PHAT* method generated a bad PDF, the error would be amplified in the SPDF, resulting in larger localization errors. This was demonstrated by the high P95 values, certainly when localizing vocal sounds. Therefore, the four array results are most accurate, but this would drastically change in larger rooms, as the overall distance of the points to the nodes would increase. Because the *dot product PHAT* method is computationally more intensive, a trade-off should be made, depending on the desired accuracy, the room size and the number of arrays in the room.

## 5. Conclusions

In this article, an acoustic localization algorithm for low-cost low-power WASNs is elaborated and characterized in a practical test setup. The sensor nodes contained a microphone array with two elements spaced 10 cm apart. To estimate the AoA, the delay-based method was selected because it is computationally most efficient. This method calculates a cross correlation function between the recorded signals of the two microphone elements. The resulting correlation points are represented as an angular PDF that is used by the matching algorithm to estimate the sound position. Two time-domain cross correlation methods were evaluated for generating the PDF: *dot product* and *dot product PHAT*. The proposed localization method generates SPDFs, based on the angular PDFs of all the nodes in the room. The highest value in the combined SPDF defines the estimated location.

The localization method was evaluated in a test setup with five nodes in a rectangular room of 4.25 m by 9.20 m. Four nodes were placed at the center of each wall, and one was placed in a corner orthogonal to the room diagonal. The tests indicated that the node placed in the corner gave inaccurate results due to the limited use of the array aperture and propagation effects in the corner of the room. When evaluating pairwise node combinations, the nodes at the shortest wall exhibited the best results for the *dot product* method, while the long walls were preferred for the *dot product PHAT* method. Adding a third array to the system reduced the mean localization errors by 37% for the *dot product* method, while adding a fourth array had no significant improvement compared to the three arrays solution. The mean absolute localization error of this method was ± 60 cm. On the other hand, *Dot product PHAT* benefited from the additional fourth array and achieved an absolute mean localization error of 40 cm localizing white noise. When vocal sounds were recorded, an increase of at least 100% in localization errors was observed.

The proposed localization method was compared to the established least squares triangulation algorithm. When localizing white noise, the matching algorithm performed 38% better for the *dot product* method and 114% better when using *dot product PHAT*. The localization of vocal sounds resulted in a small improvement in localization accuracy. The *dot product* method performed 5% better, while *dot product PHAT* achieved an accuracy improvement of 12%. These results demonstrate the effectiveness of the described matching algorithm. When the sensor nodes transmitted 21 correlation points instead of a single AoA value, significant accuracy improvements could be achieved with the proposed PDF matching algorithm.

Future work will focus on improving the accuracy by exploiting reverberations in the room, including Non-Line-Of-Sight (NLOS) effects in the predefined dataset, and using post-processing techniques (e.g., dead reckoning).

## Figures and Tables

**Table 1:** The time and energy consumption of each step in the localization process of one event on the EFM32 WASN-node.

	Time (ms)	Energy Consumption (mJ)
**Microphone array [27]**	410	0.169
**Sampling**	32	0.475
**Processing (dot product)**	6.9	0.284
**Processing (dot product PHAT)**	14.9	0.614
**Transmitting (All samples)**	128.1	124.6
**Transmitting (Correlation points)**	31.2	1.81
**Transmitting (AoA value)**	30	1.68

**Table 2:** $RT_{60}$ Test setup.

**Frequency (Hz)**	63	125	250	500	1000	2000	4000	8000
**RT60 (s)**	1.10	0.94	0.87	0.89	0.82	0.92	0.86	0.71

**Table 3:** SI assessment of the different individual arrays for the two calculation methods using WN recordings.

	*SI*		
**Setup**	**Mean**	**P50**	**P95**
	*dot product*		
**Short walls (AB)**	0.0842	0.0567	0.2813
**Long walls (CD)**	0.1050	0.0612	0.3547
**Corner—diagonal (E)**	0.3563	0.3495	0.7121
	*dot product PHAT*		
**Short walls (AB)**	0.0998	0.0572	0.3654
**Long walls (CD)**	0.1028	0.0391	0.5060
**Corner—diagonal (E)**	0.1631	0.0521	0.7706

**Table 4:** SI and ${\hat{\epsilon }}_{\mathrm{loc}}$ assessment of the different array pairs for the two calculation methods using WN recordings.

	SI			${\hat{\epsilon }}_{\mathrm{loc}}$		
**Setup**	**Mean**	**P50**	**P95**	**Mean**	**P50**	**P95**
	*dot product*					
**Short walls (AB)**	0.0263	0.0151	0.0937	0.0962	0.0568	0.3082
**Long walls (CD)**	0.0550	0.0292	0.2086	0.1228	0.0771	0.3273
**Orthogonal (AC, BD, AD, BC)**	0.0342	0.0171	0.1308	0.1021	0.0606	0.3307
	*dot product PHAT*					
**Short walls (AB)**	0.0367	0.0158	0.1440	0.1334	0.0624	0.4421
**Long walls (CD)**	0.0344	0.0164	0.1319	0.0779	0.0489	0.2319
**Orthogonal (AC, BD, AD, BC)**	0.0383	0.0119	0.1744	0.1011	0.0429	0.3673

**Table 5:** ${\hat{\epsilon }}_{\mathrm{loc}}$ and mean $\epsilon_{\mathrm{loc}}$ assessment of the matching algorithm for different array combinations, calculation methods and sounds. The highlighted rows list the best performing array combinations.

	WN				Voice			
	**${\hat{\epsilon }}_{\mathrm{loc}}$**			**$\epsilon_{\mathrm{loc}}$ [cm]**	**${\hat{\epsilon }}_{\mathrm{loc}}$**			**$\epsilon_{\mathrm{loc}}$ [cm]**
**Number of Arrays**	**Mean**	**P50**	**P95**	**Mean**	**Mean**	**P50**	**P95**	**Mean**
	*dot product*							
**2 (AB)**	0.0962	0.0568	0.3082	97	0.3340	0.2974	0.7424	338
**2 (CD)**	0.1228	0.0771	0.3273	124	0.2147	0.2125	0.4079	218
**2 (AC, BD, AD, BC)**	0.1021	0.0606	0.3307	103	0.2127	0.1984	0.4212	216
**3 (ABC, ABD)**	0.0605	0.0384	0.2071	61	0.1989	0.1742	0.4236	202
**3 (ACD, BCD)**	0.0809	0.0445	0.3059	82	0.1993	0.1895	0.3920	202
**4 (ABCD)**	**0.0576**	**0.0335**	**0.2026**	**58**	**0.1891**	**0.1748**	**0.3805**	**192**
	*dot product PHAT*							
**2 (AB)**	0.1334	0.0624	0.4421	135	0.3098	0.2691	0.6636	314
**2 (CD)**	0.0779	0.0489	0.2319	79	0.2026	0.1646	0.5183	205
**2 (AC, BD, AD, BC)**	0.1011	0.0429	0.3673	102	0.2268	0.1776	0.5930	230
**3 (ABC, ABD)**	0.0572	0.0280	0.2151	58	0.2450	0.1975	0.6270	248
**3 (ACD, BCD)**	0.0600	0.0318	0.2108	61	**0.1991**	**0.1502**	**0.5748**	**202**
**4 (ABCD)**	**0.0397**	**0.0242**	**0.1246**	**40**	0.2100	0.1636	0.5808	213

**Table 6:** ${\hat{\epsilon }}_{\mathrm{loc}}$ and mean $\epsilon_{\mathrm{loc}}$ assessment of the triangulation algorithm for different array combinations, calculation methods and sounds. The highlighted rows list the best performing array combinations.

	WN				Voice			
	**${\hat{\epsilon }}_{\mathrm{loc}}$**			**$\epsilon_{\mathrm{loc}}$ [cm]**	**${\hat{\epsilon }}_{\mathrm{loc}}$**			**$\epsilon_{\mathrm{loc}}$ [cm]**
**Number of Arrays**	**Mean**	**P50**	**P95**	**Mean**	**Mean**	**P50**	**P95**	**Mean**
	*dot product*							
**2 (AB)**	0.1898	0.1099	0.8641	192	1.2140	0.6119	5.0940	1230
**2 (CD)**	0.1878	0.1181	0.5598	190	0.8439	0.3370	4.1370	855
**2 (AC, BD, AD, BC)**	0.1673	0.0855	0.3614	170	0.2149	0.2116	0.3784	218
**3 (ABC, ABD)**	0.0874	0.0667	0.2365	89	0.2019	0.1901	0.3577	205
**3 (ACD, BCD)**	0.0981	0.0678	0.2936	99	0.2076	0.2134	0.3555	210
**4 (ABCD)**	**0.0852**	**0.0632**	**0.2629**	**85**	**0.1990**	**0.2093**	**0.3337**	**202**
	*dot product PHAT*							
**2 (AB)**	1.1700	0.4331	8.2180	1186	1.3230	0.8452	5.1530	1341
**2 (CD)**	1.4530	0.0660	17.900	1473	0.4230	0.2563	2.1850	429
**2 (AC, BD, AD, BC)**	0.8631	0.0795	2.3080	875	0.8681	0.2963	3.8430	880
**3 (ABC, ABD)**	0.2941	0.1677	1.2060	298	0.4073	0.2451	1.3070	413
**3 (ACD, BCD)**	0.1605	0.0728	0.4562	168	0.2653	0.2149	0.6278	269
**4 (ABCD)**	**0.1443**	**0.1228**	**0.4086**	**146**	**0.2367**	**0.2095**	**0.5189**	**240**

## Abbreviations

The following abbreviations are used in this manuscript:

AAD	Acoustic Activity Detector
AoA	Angle of Arrival
GCC-PHAT	Generalized Cross Correlation with PHAse Transform
GEVD-MUSIC	Generalized EigenValue Decomposition-MUSIC
LOS	Line-Of-Sight
LP	Linear Prediction
MUSIC	MUltiple SIgnal Classification
P50	50th percentile
P95	95th percentile
PDF	Probability Density Function
SI	Surface Interval
SPDF	Spatial Probability Density Function
SRP	Steered-Response Power
TDoA	Time Difference of Arrival
WASN	Wireless Acoustic Sensor Network
WN	White Noise
WSN	Wireless Sensor Networks

## References

[1] Tubaishat M., Madria S. (2003). Sensor networks: An overview. IEEE Potentials.

[2] Gomez C., Paradells J. (2010). Wireless home automation networks: A survey of architectures and technologies. IEEE Commun. Mag..

[3] Yanco H.A., Haigh K.Z. (2002). Automation as caregiver: A survey of issues and technologies. Am. Assoc. Artif. Intell..

[4] Crocco M., Cristani M., Trucco A., Murino V. (2016). Audio Surveillance: A Systematic Review. ACM Comput. Surv..

[5] Shah M., Javed O., Shafique K. (2007). Automated Visual Surveillance in Realistic Scenarios. IEEE MultiMedia.

[6] Cobos M., Antonacci F., Alexandridis A., Mouchtaris A., Lee B. (2017). A Survey of Sound Source Localization Methods in Wireless Acoustic Sensor Networks. Wireless Commun. Mob. Comput..

[7] Deng F., Guan S., Yue X., Gu X., Chen J., Lv J., Li J. (2017). Energy-Based Sound Source Localization with Low Power Consumption in Wireless Sensor Networks. IEEE Trans. Ind. Electron..

[8] Kupper A. (2005). Location-Based Services: Fundamentals and Operation.

[9] Casilari E., Cano-García J.M., Campos-Garrido G. (2010). Modeling of Current Consumption in 802.15.4/ZigBee Sensor Motes. Sensors.

[10] Brandstein M., Ward D. (2001). Microphone Arrays: Signal Processing Techniques and Applications.

[11] Dostálek P., Vašek V., Dolinay J. (2009). Direction of Sound Wave Arrival Determination Using Time-delay Estimation and Beamforming Methods. WSEAS Trans. Circuits Syst..

[12] Dmochowski J., Benesty J., Affes S. (2007). A Generalized Steered Response Power Method for Computationally Viable Source Localization. IEEE Trans. Audio Speech Lang. Process..

[13] DiBiase J.H., Silverman H.F., Brandstein M.S. (2001). Robust Localization in Reverberant Rooms.

[14] Zhao Y., Chen X., Wang B. (2013). Real-time sound source localization using hybrid framework. Appl. Acoust..

[15] Nakamura K., Nakadai K., Asano F., Ince G. Intelligent Sound Source Localization and its application to multimodal human tracking. Proceedings of the 2011 IEEE/RSJ International Conference on Intelligent Robots and Systems.

[16] Benesty J., Chen J., Huang Y., Dmochowski J. (2007). On Microphone-Array Beamforming From a MIMO Acoustic Signal Processing Perspective. IEEE Trans. Audio Speech Lang. Process..

[17] Astapov S., Preden J.S., Berdnikova J. Simplified acoustic localization by linear arrays for wireless sensor networks. Proceedings of the 2013 18th International Conference on Digital Signal Processing (DSP).

[18] Savic V., Larsson E.G. Fingerprinting-Based Positioning in Distributed Massive MIMO Systems. Proceedings of the 2015 IEEE 82nd Vehicular Technology Conference (VTC2015-Fall).

[19] Munoz D., Lara F., Vargas C., Caldera R. (2009). Position Location Techniques and Applications.

[20] Van Den Broeck B., Bertrand A., Karsmakers P., Vanrumste B., Van hamme H., Moonen M. Time-domain generalized cross correlation phase transform sound source localization for small microphone arrays. Proceedings of the 2012 5th European DSP Education and Research Conference (EDERC).

[21] Knapp C., Carter G. (1976). The generalized correlation method for estimation of time delay. IEEE Trans. Audio Speech Lang. Process..

[22] Wielandt S., De Strycker L. (2017). Indoor Multipath Assisted Angle of Arrival Localization. Sensors.

[23] Gay S.L., Benesty J. (2000). Acoustic Signal Processing for Telecommunication.

[24] Compagnoni M., Bestagini P., Antonacci F., Sarti A., Tubaro S. (2012). Localization of Acoustic Sources through the Fitting of Propagation Cones Using Multiple Independent Arrays. IEEE Trans. Audio Speech Lang. Process..

[25] Ciuonzo D., Rossi P.S. (2014). Decision Fusion With Unknown Sensor Detection Probability. IEEE Signal Process. Lett..

[26] Badami K., Lauwereins S., Meert W., Verhelst M. 24.2 Context-aware hierarchical information-sensing in a 6 uW 90 nm CMOS voice activity detector. Proceedings of the 2015 IEEE International Solid-State Circuits Conference (ISSCC).

[27] Thoen B., Ottoy G., De Strycker L. An ultra-low-power omnidirectional MEMS microphone array for wireless acoustic sensors. Proceedings of the 2017 IEEE SENSORS.

[28] Thoen B. (1969). IEEE Recommended Practice for Speech Quality Measurements. IEEE Trans. Audio Electroacoust..

